# The Hospital World

**Published:** 1911-02-04

**Authors:** 


### The Hospital World.

### Personalia.

The King has consented graciously to become patron of the Royal Eye Infirmary, Plymouth, in succession to his late Majesty.

Mr. John Pye, speaking at the half-yearly meeting of the Ipswich and East Suffolk Hospital, recalled very aptly the life work of the late Mr. F. W. Canham, the founder of the Hospital Sunday movement at Felixstowe. He was, as Mr. Pye remarked, "one of the hardest working men our hospital has ever had." Mr. H. W. Mason, who was in the chair, followed with the remark that Mr. Canham's enthusiasm in inspiring collections was extraordinary. This is a rare gift, which is perfectly invaluable when added to the more workaday qualities of the routine worker, and the combination of the two in one man is not found too often.

The following record of a striking personality is taken from the annual report of the Budleigh Salterton Cottage Hospital: "The institution has sustained a great loss by the death of Dr. Brushfield, who was so closely associated with it from the very first. It was mainly owing to his energy that the scheme for erecting a Cottage Hospital as a Jubilee memorial to the late Queen Victoria was carried to a successful issue. He was chairman of the Building Committee, and assumed the chairmanship of the Hospital Committee at its opening, and held that post for many years. On the death of the Rev. J. Boucher, he was appointed Vice-President. He was one of the original trustees, and was also one of the honorary consulting physicians, and was ever ready to give his valuable advice and counsel. His geniality and kindness of heart will live in the remembrance of all who came in contact with him." For those who can read between the lines this short account contains a story of personal service that it has been the great achievement of the voluntary system to awaken.

Lord Winterstoke's recent gift of £5,000 towards the cost of the contemplated extension of the Bristol General Hospital is another proof of the interest which his lordship takes in the Bristol charities. For many years the firm of Messrs. W. D. & H. O. Wills, in which his father and uncle were partners before him, had their chief warehouse and factory in Redcliff Street. This is but three or four minutes' walk from the Bristol General Hospital, and Lord Winterstoke's nephew, Mr. George Alfred Wills, to whose generosity Bristol citizens are indebted for the preservation of a portion of the Leigh Woods, has for many years taken an official position on the committee. It was Mr. Wills who purchased these woods and handed them over to be kept in trust for the benefit of the city, and he has always supported the hospital, the past records of which for public benefit are such as to rank it among those most deserving and popular of the Bristol institutions. Bristol has good reason to be proud of Lord Winterstoke, whose liberality, with that of other members of the Wills family, has done much for the city and its charities.—Since the above was written news comes of Lord Winterstoke's sudden death last Sunday, which, his advanced age notwithstanding, was quite without warning. On the previous day he was apparently perfectly well and attended a parade of horses entered for forthcoming shows. His niece was called at 3 a.m. next morning, and two hours later he died. Lord Winterstoke was the youngest son of Mr. William Day Wills, one of the founders of W. D. and H. O. Wills, tobacconists. He was born in 1830. He gave up the Bar for the tobacco industry at Bristol, and was elected Liberal M.P. for Coventry in 1880. He sat till 1885, and from 1894 to 1900 represented South Bristol, of which city he became Sheriff :. He received a baronetcy in 1902, a peerage in 1905, and his industrial reputation was established by the activity he showed in forming the great combine known as the Imperial Tobacco Company. In 1901 this absorbed 13 smaller companies, the interests of which it purchased for £11,957,000. He had been a director of the Great Western Railway, and actually was a founder of the London Missionary Society. Bristol owes to him its art gallery, £35,000 towards the Foundation Fund of its university, a statue of Edmund Burke, and several of the organs in its churches. Above we have noted his work for Bristol Hospitals. No. heir succeeds him.

Mr. Alderman John Berry, J.P., a well-known citizen of Bolton, has died at the age of seventy-two. A man who, in the old phrase, rose from the ranks, he possessed not only the faculty for making success, but a personality which expressed itself in very individual ways. For instance, the interest which he took in instructing boys in machine-making matters developed into the present Technical Instruction Committee now supported by the Bolton Education Committee. He was a keen organiser, and the Bolton Infirmary has gained by his powers in this direction no smaller sum than £70,000, for this vast sum represents the total raised up to the present by the Committee of the Working Men's Hospital Saturday Fund. Mr. Berry thus introduced two great movements, technical instruction and working men's collections, into his factories, and was a man who gave his best brains to the personal service he rendered.

### Elections and Appointments.

Mr. C. C. Sibthorp has been re-elected treasurer of the Lincoln County Hospital.

Mr. Thomas J. Atkinson has been elected a life member of the Royal Victoria Eye and Ear Hospital, Dublin.

Messrs. Hurry, Duy & Co., of Worcester, have been appointed auditors for the year to the Worcester General Infirmary.

Mr. Councillor J. G. Frost and Mr. Councillor Turner have been elected governors of the Macclesfield Infirmary.

Mr. E. A. Oliver, chairman of the Ipswich Employees' Committee, has been elected a life governor of the East Suffolk and Ipswich Hospital.

The Vice-Chairman of the Board of Governors, Sir Arthur Grey Hazlerigg, Bart., has been appointed a trustee of the Leicester Infirmary.

The Rev. W. H. Thornton, Dr. Mapleton, and the Rev. A. Caldecott have been re-elected to the committee of management of the Moreton Hampstead Cottage Hospital. Canon Palmes, the recently-appointed vicar of Lustleigh, has been invited to fill the place of the Rev. W. Gordon Baillie, Mr. E. J. Cuming has been re-elected hon. secretary, and Mr. H. Knight treasurer.

The following names have been added to the list of Life Governors of the Leicester Infirmary: Major Andrew Coats, D.S.O., in recognition of a generous gift of £100; Mr. Kenneth McAlpin, in appreciation of the recent effort of the Leicester Football Club, which produced £115; Miss Ivy Pick, for the several entertainments organised by her; Mr. William Warrington Harding and Mr. Ernest Warrington Harding, in respect of a gift of £105 in memory of their father, the late Mr. William Harding; and Mrs. L. A. Doudney, the nominee of the executors of the will of the late Mrs. M. A. Jones.

